# MixtureTree Annotator: A Program for Automatic Colorization and Visual Annotation of MixtureTree

**DOI:** 10.1371/journal.pone.0118893

**Published:** 2015-03-31

**Authors:** Shu-Chuan Chen, Aaron Ogata

**Affiliations:** 1 Department of Mathematics, Idaho State University, Pocatello, Idaho, United States of America; 2 School of Computing, Informatics, and Decision Systems Engineering, Arizona State University, Tempe, Arizona, United States of America; 3 School of Biological and Health Systems Engineering, Arizona State University, Tempe, Arizona, United States of America; New York University School of Medicine, UNITED STATES

## Abstract

The MixtureTree Annotator, written in JAVA, allows the user to automatically color any phylogenetic tree in Newick format generated from any phylogeny reconstruction program and output the Nexus file. By providing the ability to automatically color the tree by sequence name, the MixtureTree Annotator provides a unique advantage over any other programs which perform a similar function. In addition, the MixtureTree Annotator is the only package that can efficiently annotate the output produced by MixtureTree with mutation information and coalescent time information. In order to visualize the resulting output file, a modified version of FigTree is used. Certain popular methods, which lack good built-in visualization tools, for example, MEGA, Mesquite, PHY-FI, TreeView, treeGraph and Geneious, may give results with human errors due to either manually adding colors to each node or with other limitations, for example only using color based on a number, such as branch length, or by taxonomy. In addition to allowing the user to automatically color any given Newick tree by sequence name, the MixtureTree Annotator is the only method that allows the user to automatically annotate the resulting tree created by the MixtureTree program. The MixtureTree Annotator is fast and easy-to-use, while still allowing the user full control over the coloring and annotating process.

## Introduction

The Newick tree format [1] is used in many scientific disciplines, with a major role in reconstructive phylogeny. The format is relatively simple and provides the ability to show the relative distance and relationship between leaves (i.e., operational taxonomic units, OTUs); however, it lacks the ability for the user to add color and annotations to each branch. In reconstructive phylogeny, it is important to be able to show clusters of leaves and to provide annotations such as mutation information, especially when the sample size is large. The MixtureTree Annotator allows the user to automatically color any given Newick tree generated by many popular software packages, including but not limited to MixtureTree [2], MEGA [3], MrBayes [4], and SeaView [5]. By providing the ability to automatically color the tree by sequence name, the MixtureTree Annotator provides an advantage over other current programs. For example, MEGA [3], Mesquite [6], PHY-FI [7], TreeView [8], and Geneious [9], the most popular programs that allow the user to add color to a Newick tree, require the user to manually add color to each node; this can easily result in repetitive clicking with a high potential for human error. TreeGraph [10] allows the user to automatically color a tree to some extent, but it can only color based on a number, such as branch length. PhyloView [11] also allows the user to color the tree automatically by taxonomy, but requires the dataset to be named in a more specific manner. In contrast to the above programs, the MixtureTree Annotator allows the user to easily assign user-defined colors to different groups of sequences that have commonalities such as source population or phenotypic character state. In addition, MixtureTree Annotator is the only program available that can properly annotate the output produced by the MixtureTree [2, 14, 15, 16] with mutation information and coalescent time information. However, trees that are not generated by the MixtureTree package cannot be annotated at this time. A program that provides similar coloring abilities to MixtureTree Annotator is ColorTree [12], but it does not provide annotation abilities.

## Material and Methods

**Fig 1:**
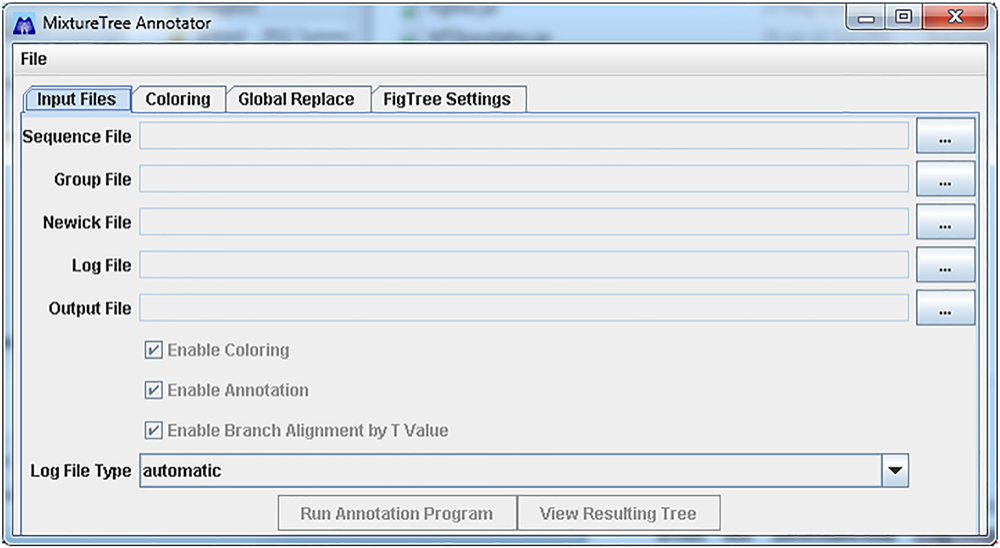
Main Screen of MixtureTree Annotator.

The MixtureTree Annotator can accept either a single Newick Tree File as input, or the entire output dataset of MixtureTree [2]. As illustrated in the main screen, Fig 1, there are five different types of files which may be entered. All features of this program require a Newick tree file as input, and for the user to specify an output file. In order to provide annotation ability, this program requires two input files: the sequence file and the log file. Colorization ability can be enhanced by providing a file for group definitions. The sequence file contains a list of sequence names, nucleotide data and frequencies. The log file, generated by the MixtureTree algorithm, contains the debugging output. The group file contains a list of group names and their sequence members. These files are described in further details in the User Guide. The user may specify if he or she would like to add color, annotation information, or both to the resulting tree. The generated output file from the MixtureTree Annotator is a modified form of the Nexus format. In order to visualize the output file from this program, a modified version of the program FigTree [17] must be used.

## Results

In order to demonstrate the utility of this program in a real-life application, a sample dataset from the International HapMap Project is used. It includes data from two human population groups: the Yoruba people from Nigeria, Africa (YRI) and the U.S. European American group (Eu_Am) [13]. There are 52 sequences used in this dataset, with 34 from Eu_Am and 18 from YRI. One hundred sites were taken from Chromosome 1 between 3290990 and 3498109 of HapMap Phase 3 Release 2, NCBI Build 36. The resulting tree was generated by MixtureTree v3.0 [2,15] using the modalEM algorithm with a sliding scale (p) value of 0.001 [2,14, 15, 16]. The discussion is divided into two sections: Newick Tree Colorization and Newick Tree Annotation and entering external files.

### Newick Tree Colorization

**Fig 2:**
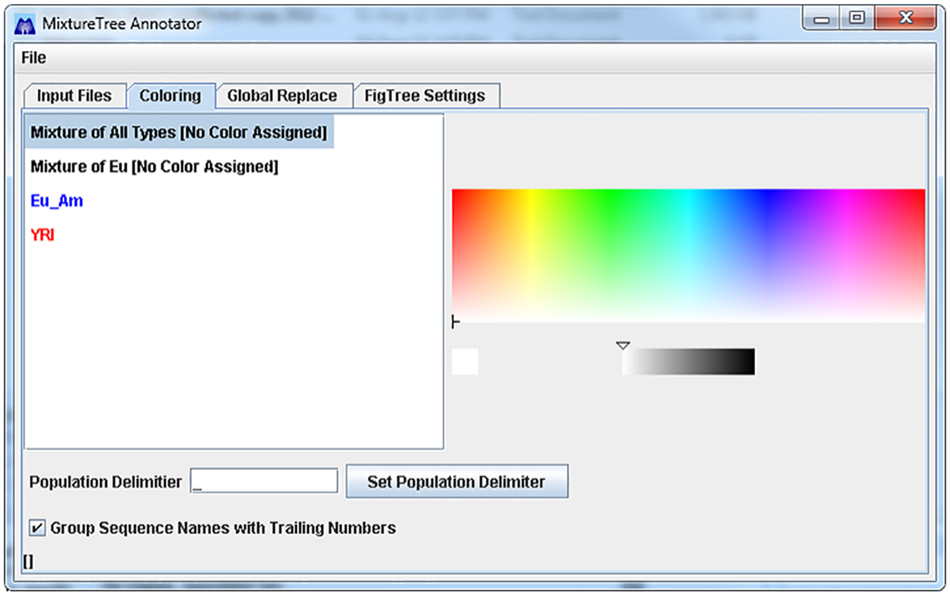
Default Coloring Page of MixtureTree Annotator.

**Fig 3:**
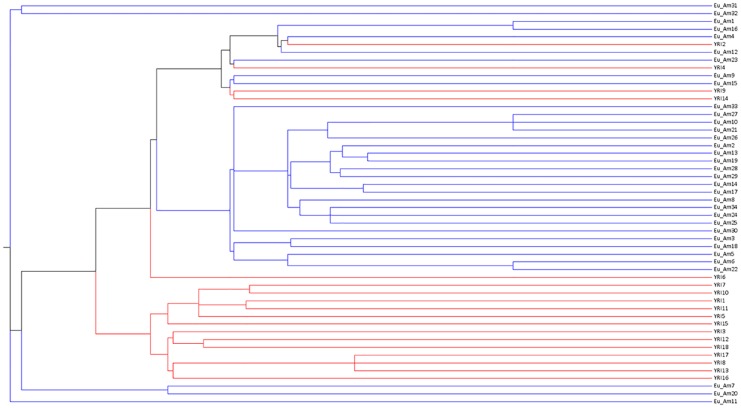
Resulting Hapmap Tree.

Colorization in Newick trees is important because it enables the user to quickly and accurately visualize clusters of DNA sequences, especially when the sample size is large. A typical coloring screen is shown in Fig 2, in which the different lineages are listed on the left and the color picker is on the right. Based on user preference, the YRI population group could be colored red and the Eu_Am population group colored in blue. A Newick tree normally consists of a single, solid black color. However, as shown in Fig 3, the benefits of tree colorization are clear. One immediately notices two large distinct clusters, namely the distinct Eu_Am and YRI groups. The advantage of this package is that by clicking on the color picker, the user can easily assign a color to each group or sequence.

### Newick Tree Annotation

**Fig 4:**
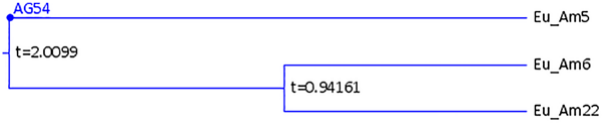
Enlarged Selection from Hapmap Tree.

Annotation of phylogenetic trees is important for estimating the actual ancestral sequences and the evolution time. The Log file generated by MixtureTree records every change of nucleotide over time. The MixtureTree Annotator could help to display when mutations happen in xy### format where x is the ancestral nucleotide at time t+ϵ, y is the mutated ancestor type at time t, and ### is the site at which the mutation occurred. Fig 4 shows an example where the currently observed nucleotide at site 54 of Eu_Am5 is a G, and it is an A in the ancestral sequence at site 54 when time t = 2.009. The MixtureTree algorithm [2, 14, 15, 16] constructs the tree in a reverse time manner. The currently observed sequences are given by time t = 0. The most recent common ancestor is located at far left.

The case at a given time point t is shown in Table 1. The mutation information for this case is AT1. That is at time t+ ϵ, where ϵ>0, the nucleotide of site 1 mutates from A to T. The merge time information is in a self-explanatory format. The time scale used is described in Chen and Lindsay, 2006 [16]. For further clarification, AG54 in Fig 4 shows that at time t = 2.0099, the common ancestral sequence of Eu_Am5, Eu_Am6, and Eu_Am22 at site 54 has nucleotide A. At time t = 0, the sequence of Eu_Am5 at site 54 has nucleotide G. This is because the mutation (at time = 2.0099, site 54, from A to G) makes Eu_Am5 become a distinct lineage. In this specific example, Eu_Am6 and Eu_Am22 actually contain the same genetic sequences.

**Table 1:** Demonstration of Annotation Format.

Sequence Location	1	2	3	4
Nucleotide at time t+ϵ	A	C	T	G
Nucleotide at time t	T	C	T	G
**Mutation Information**	AT1			

### Entering external files: An example using Newick tree files generated from Mixture Tree

**Fig 5:**
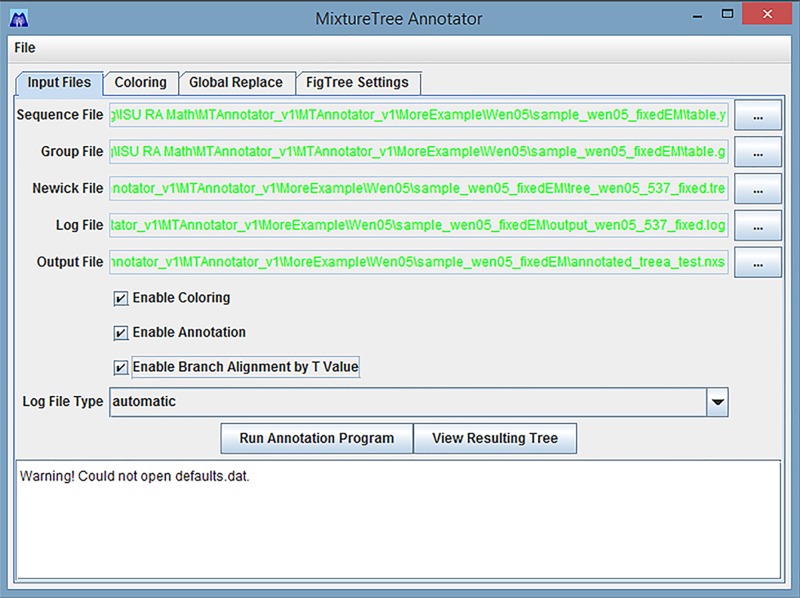
An Example of Entering External Files Generated from MixtureTree.

In Fig 5, the sequence file (filename.y) and group file (filename.g) can be generated from the table converter. The table converter, one supplementary package included in MixtureTree, converts the sequence format into MixtureTree input format.

The MixtureTree generates tree file in Newick format (filename.tre) and Log file (filename.log) with mutation information at the given time back to the ancient state. The tree file and log file can be placed directly into 'Newick File' selection bar and 'Log File', respectively.

The output of MixtureTree Annotator is Nexus format with all the color and annotating information. The name and directory of output file can be assigned by users.

### Entering external files: An example using simple Newick tree files generated from other packages

**Fig 6:**
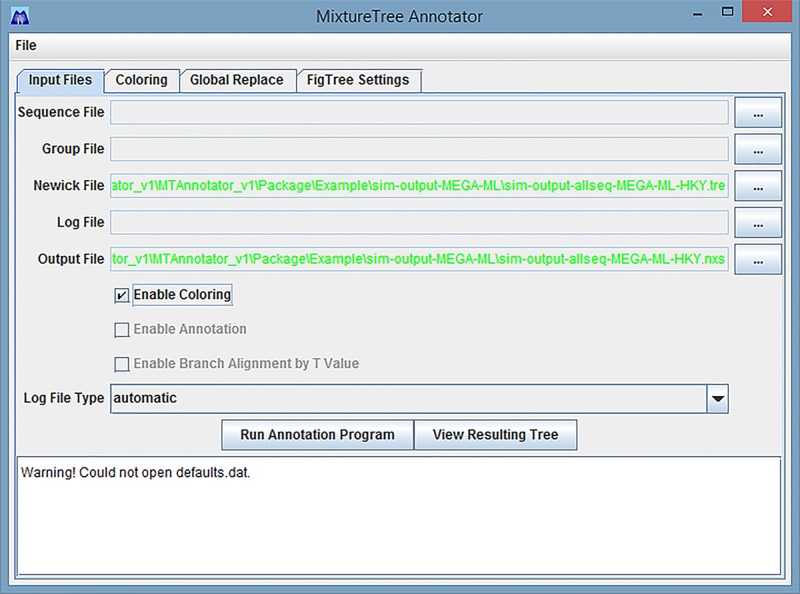
An Example of Entering External Files Generated from MEGA 6.

**Fig 7:**
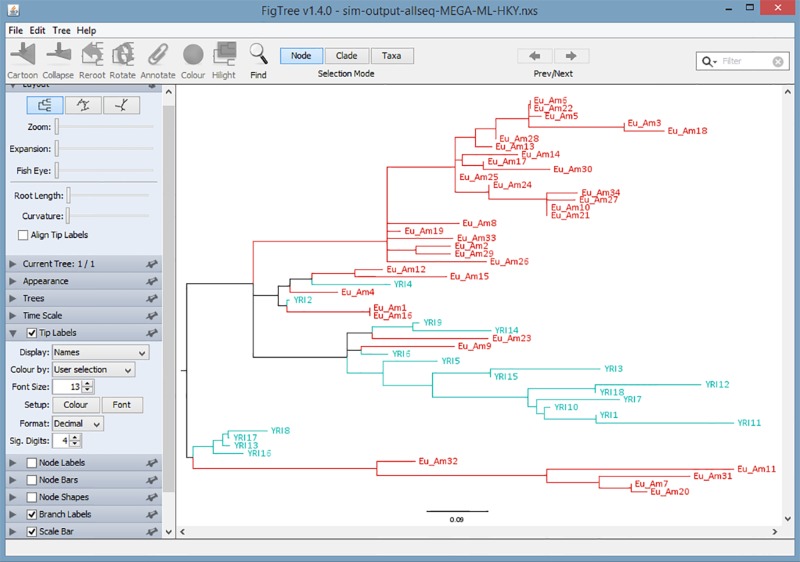
The Tree Resulting from using external files generated by MEGA 6.

Users also can input the Newick file from any phylogeny reconstruction program and output the *.nxs file. We use MEGA 6 [27] to reconstruct the phylogeny of one example data set. Next, we save and export the phylogenetic tree into Newick format and upload the Newick file that is generated by MEGA into 'Newick File' file selection bar. In Fig 6, even though there is no sequence file, group file and log file, MixtureTree Annotator can still generate the colorized tree when users input the Newick file generated from other programs. The resulting tree is shown in Fig 7.

### Distances Supports

**Fig 8:**
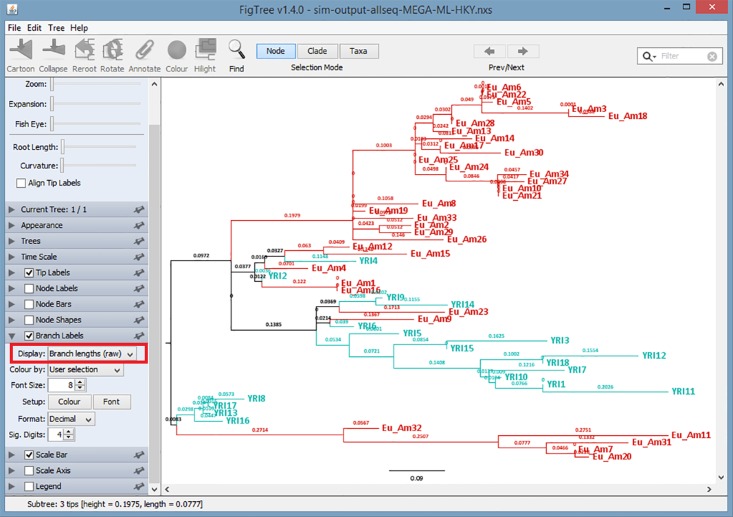
An Example of Distances Support in the Viewer.

The branch lengths (either internal or external) represent the distance information for the time that merged the two sequences. The distance information is written in the Newick file generated by MixtureTree or other packages. The display of distance could be edited by the function panel in FigTree by clicking the following: 'Branch Labels' >'Branch lengths (raw)' in' Display'; a pull-down menu is shown in Fig 8.

## Discussion and Conclusions

The unique advantage of MixtureTree Annotator is that it is the only package that can easily and efficiently annotate the output produced by MixtureTree with mutation information and coalescent time information. Table 2 compares MixtureTree Annotator with other active tree visualization tools, including Dendroscope [23], HyperTree [19], NJPlot [26], HyperGeny [20], CTree [18], and BAOBAB [21]. These useful tree drawing, editing and manipulation tools can generate the topology with subtree collapse, re-rooting, rotating, adding/removing the taxa and colorizing them individually in different layouts (rectangular, slanted, circular views). Some packages also annotate information such as branch lengths, confidence values and summary of subtrees on the internal nodes. Phylowidget [24] and Archaeopteryx [22] include the annotating and tree handling functionalities, which are relatively comprehensive tools. The tree viewer iTOL [25], TreeIllustrator [28], and Archaeopteryx [22] also integrate the taxonomy of organisms into the package, which allows users to compare and identify new data with the known classification of organisms. Although MixtureTree Annotator does not incorporate taxonomic information, it does integrate the functions of FigTree (http://tree.bio.ed.ac.uk/software/figtree/) that has both annotation and tree manipulation as other packages have. Its colorization can easily define the color of a group by information given in the sequence name in the graphic interface. Its functionality is more intuitive than other published packages. Another distinctive and essential feature in MixtureTree Annotator is the mutation events annotation. The mutation estimation comes from MixtureTree’s phylogeny reconstruction package, and it could tell users the specific time and site of mutation occurrences in a sequence. MixtureTree Annotator is currently the only package available that illustrates this information on the topology tree. These features help researchers better interpret phylogeny and make hypotheses from the relationships and clusters of taxa, and convey their ideas to readers more efficiently.

**Table 2:** The Difference between MixtureTree Annotator and Other Active Visualization Tools.

Tool	Availability	Supported format	Colorization	Mutation occurred at given time	Export	Layouts	Advantage	Disadvantage
Dendroscope	Free for academic use	NeXML, Nexus or Newick format	Assign color individually (Edit>Format)	N/A	.eps,. svg,. png,. jpg,. gif,. bmp,. pdf	Rectangular, slanted, circular and radial views	1. Supports general functionalities for tree. algorithms, subtree collapse, coloring and re-rooting. 2. Shows tanglegram for two trees or networks.	Not favorable for users to assign color based on a group of sequences
iTOL (interactive tree of life)	Online version, free	Newick, Nexus or PhyloXML	No graphic interface of colorizing function for branches and nodes	N/A	.svg,. png,. eps,. pdf	Rectangularand circular-ring views	1. Is flexible for users who need to construct a phylogeny based on well-known organisms with NCBI taxonomy IDs. 2. Clade could be collapsed by setting proportion of distance.	View online only; Not favorable for users to assign the color of subtrees or a group of sequences
HyperTree	Free for academic use	PHYLIP, phyloXML, Newick	Assign color individually by graphic inferface (Edit>Color) or color editing file	N/A	PHYLIP format	Linear, radial, unrooted views	1. Contains simple interface and functionalities for users to draw and colorize a phylogeny 2. Users can re-root the phylogeny by selecting a given node	Lack of important information in phylogenetics such as branch length, confidence value, node annotation
NJPlot	Free for academic use	Newick	N/A	N/A	.pdf and. eps	Rectangular	Tree operations, including new outgroup assignment, rotation and subtree visualization, are useful for phylogenic display	Limited functionality and not recommended for large datasets
HyperGeny	Free for academic use, online and downloadable packages are both available	Newick	Can only assign one color for all nodes	N/A	Newick format	Unrooted view	1. Can collapse the subtree and output the Newick file from chosen node. 2. Is available for large tree structures and datasets	Only for hyperbolic visualization
CTree	Free	Newick	Can assign color to selected subtree	N/A	.pdf, Newick format	Rectangular and unrooted views	1. Has simplistic interface for user to generate a publishable tree along with distance, bootstrapping value, re-rooting and subtree colorization. 2. Comes with statistical analysis, such as calculation of subtype diversity ratio and subtype diversity variance distribution.	1. Lacks ability to handle large datasets. 2. Supports the input and output format only in Newick format and pdf file, respectively
Phylowidget	Free, online and downloadable packages are both available	Newick, NHX and Nexus	Assigns color individually by graphic interface	N/A	.jpeg,. pdf,. png	Rectangular, slanted, circular and unrooted trees	1. Has comprehensive visualization tool for reconstructing phylogenetic trees. 2. Can handle big datasets	1. Takes more time and memory to run the program. 2. Lacks intuitive color assignment for sequences
BAOBAB	Free for academic use	Newick, Nexus or XML format	Can assign color for subtree based on attributes of sequence name	N/A	Newick, XML and Pag	Rectangular and unrooted trees	1. Can add, delete or modify the node and/or tree structure. Is useful in generating an artificial tree to illustrate user ideas. 2. Color selection is useful for users who are familiar with color manager function.	1. Is not very intuitive in assigning the color of nodes and branches. 2. Has simple interface but lacks functionality, such as different tree layouts, topology algorithms
TreeIllustrator	Free for academic use	Newick, NEXUS	N/A	N/A	.ps and. jpeg	Phylogram, rectangular, radial and slanted cladograms	1. Has built-in tree of life browser and search engine. 2. Customizes phylogenetic trees and compares them with the current classification of organisms.	Lacks annotating functionalities for sequence, such as branch length, nodes and confidence values
Archaeopteryx	Free for academic use	New Hampshire, Nexus, ToL response XML, or (deprecated) NHX format	Color assigned in XML format	N/A	Newick, PHYLIP,. pdf,. gif,. jpeg,. png,. bmp	Rectangular, unrooted, and circular trees	1. Displays features based on taxonomy. 2. Can show node events with size of subtree, number of taxonomies and duplications.	Lacks graphic interface of color function for branches and nodes
MixtureTree Annotator	Free for academic use	Newick format	Can easily assign the color based on the name of sequence in the color function	Can annotate where and when the nucleotide substitution occurred in given sequence on the tree structure	NEXUS, Newick format; graphic output including. eps,. pdf,. jpeg,. png,. emf,. gif,. raw,. bmp,. ps	Rectangular, polar, radial trees	1. Easily incorporates colorization and mutation annotating function into FigTree phylogeny viewer. 2. Has intuitive interface for users to assign color to nodes and branches; automatically groups the sequences based on the names of sequences. 3. Has accessible view of every mutation event on tree layouts	1. Cannot collapse clade 2. Cannot integrate the taxonomy of organisms based on known classifications

From the sample dataset above, it is clear how the MixtureTree Annotator is useful to both users of MixtureTree, as well as to users who want an enhanced visualization of any general phylogenetic trees. The MixtureTree Annotator is a colorization and annotation program that is designed to assist the user when visualizing phylogenetic trees. It gives the user fine-grained control over the different settings while remaining easy-to-use. By using this program, a much clearer picture can be formed of the ancestral lines represented by different trees.

One of the more useful features of this package is easy colorization of groups based on names of sequences, and the presentation of the ancient states of nucleotides. MixtureTree Annotator generates one Nexus file for FigTree to present the colors and ancient states that are annotated by MixtureTree Annotator. All other built-in functions in FigTree are incorporated in MixtureTree Annotator, so users can easily generate the layouts of trees (circular, radial etc.) and export the graphs in different graphic formats that are contained in FigTree. MixtureTree Annotator shows every change in nucleotides over time. There is no other current interface that can manually modify or deactivate changes that users do not want to see.

## Availability and Requirements

The MixtureTree Annotator binary, source code, and S1 User Guide are available at the link http://www.mixturetree.net. It is a platform independent, Java-based program that requires Java 1.6 or higher. It implements a method of Newick tree colorization and provides visual annotation for MixtureTree. Anyone who uses this program is requested to cite the MixtureTree website and this paper.

## Supporting Information

S1 User Guide User Guide. (DOCX)
