# Supplementary material for: MixtureTree Annotator: A Program for Automatic Colorization and Visual Annotation of MixtureTree
Source: PLoS One. 2015 Mar 31;10(3):e0118893. doi: 10.1371/journal.pone.0118893 (PMC4380466; doi:10.1371/journal.pone.0118893)
Supplement: S1 User Guide — (DOCX) [file pone.0118893.s001.docx]

***MixtureTree Annotator v1.0***

***User Guide***


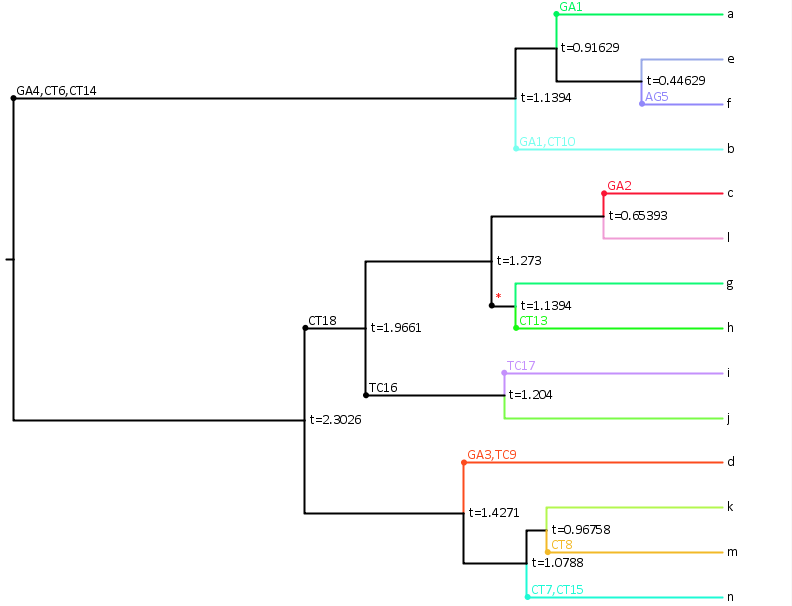


Copyright

MixtureTree Annotator: A program for automatic colorization and visual annotation of MixtureTree

Copyright (C) 2013 Shu-Chuan Chen

This program is free software: you can redistribute it and/or modify it under the terms of the GNU General Public License as published by the Free Software Foundation, either version 3 of the License, or (at your option) any later version.

This program is distributed in the hope that it will be useful, but WITHOUT ANY WARRANTY; without even the implied warranty of MERCHANTABILITY or FITNESS FOR A PARTICULAR PURPOSE. See the GNU General Public License for more details.

You should have received a copy of the GNU General Public License along with this program. If not, see <http://www.gnu.org/licenses/>.

http://www.mixturetree.net/

E-mail: scchen@isu.edu

About this Program

The program MixtureTree Annotator v1.0 provides the ability to colorize all different types of Newick trees as well as the ability to annotate the trees produced by MixtureTree (Chen *et al.* 2011a) with detailed mutation information. The program is written in Java and requires Java 1.6 or higher in order to run. The resulting tree is viewable by using a modified version of FigTree (Rambaut, 2007) [see Appendix C]. The Java 1.6 or higher is available from <http://www.oracle.com/technetwork/java/javase/downloads/index.html>.

Step 1: Program Preparation

The main directory of the downloaded MixtureTree Annotator package is depicted in the figure below.


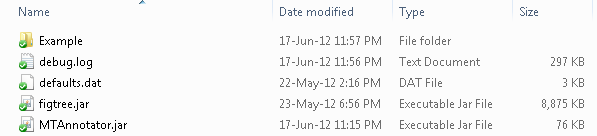


Each file or folder is described in the table below.

| **File Name** | **Description** |
| --- | --- |
| “Example” | This folder contains the data set which will be demonstrated in this User Guide. |
| Debug.log | This file contains debugging information required for the development of this program. **Users should not modify or remove this file.** |
| Defaults.dat | This file contains the default settings used for this program. **Users should not modify or remove this file.** |
| Figtree.jar | This is an executable file for the customized version of FigTree used in this package. |
| MTAnnotator.jar | **This is the main program.** Most users only need to click on this file to run this package. |

**To run this program, first find the file titled “MTAnnotator.jar” and double-click it to run it.**

Step 2: Data Input

There are two main methods of selecting the files for input. The minimum requirement for colorization is the Newick File and Output File. For annotation, a Sequence File and a Log File are also required. A Group File is optional in all cases, but will provide for more-specific colorization ability.

**Method 1: File -> Open Folder…**


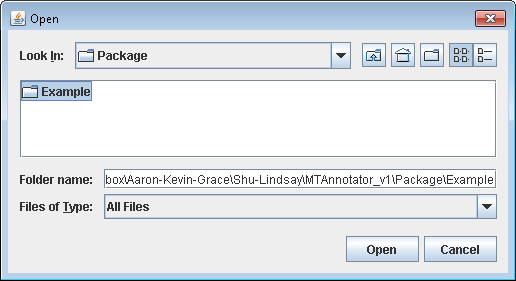


As an example, we will select the “Example” folder. The program will attempt to automatically detect the files in the folder. If more than one file is detected as being a certain type, the field is left blank and the user will have to manually input the file name and location by clicking on the […] button. **Most users will use this method to open a data set.**

**Method 2: Manual Selection**

The user is also able to manually select the files for input by using the […] button next to each field.


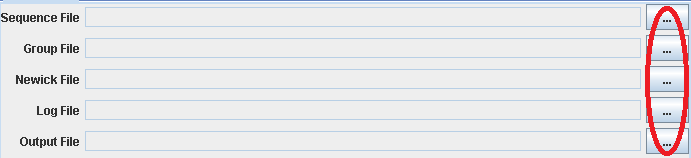


If the user wishes to manually select the Example files, the following files need to be selected.

| **File Type** | **File Name** |
| --- | --- |
| Sequence File | Example\sim-output.y-modalEM-0.001-0-sequences.dat |
| Group File | LEAVE BLANK |
| Newick File | Example\sim-output.y-modalEM-0.001-0-tree.tre |
| Log File | Example\sim-output.y-modalEM-0.001-0-log.txt |
| Output File | Example\annotated_tree.nxs |


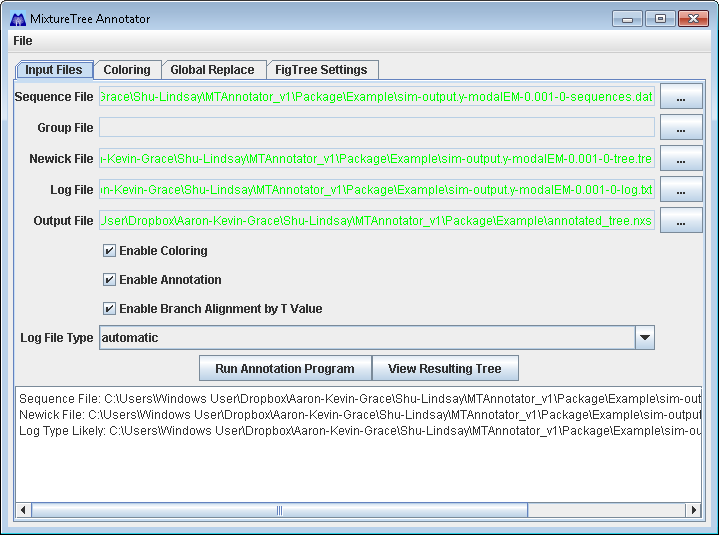


Regardless of the method utilized to open the data set, the resulting screen should look similar to the above figure if the data set was opened properly. If any entry is red, the file was detected as being invalid for that field and the program will not be able to run until the condition is corrected.

| **Sequence File** | This file contains the sequence data for this program.  Format: <Sequence Name> <Sequence Data> <Frequency of Occurrence> |
| --- | --- |
| **Group Membership File** | This file contains the group definitions.  Format: <group name>=<member>,<member>,…, |
| **Newick File** | This file contains the generated Newick Tree. The data should be in standard Newick format.  Example: (A:0.1,B:0.2,(C:0.3,D:0.4):0.5); |
| **Log File** | This file contains the generated EM algorithm output file. There are two supported types of log files which are detailed below in Log File Type.  No Example Provided |
| **Log File Type** | (automatic, mixturetree, extendedmodel, mif)  Automatic – Try to automatically determine what type the given log file is. If a type cannot be determined, “extendedmodel” is chosen.  MixtureTree – Log file generated by Bernoulli Mixture Models (MixtureTree v3.0 Only)  ExtendedModel – Log file generated by Extended Models (MixtureTree v3.0 Only)  mif – Log file generated by both models (MixtureTree v3.1 Only) |

**Further information about file types can be found in Appendix C – File Format Descriptions.**


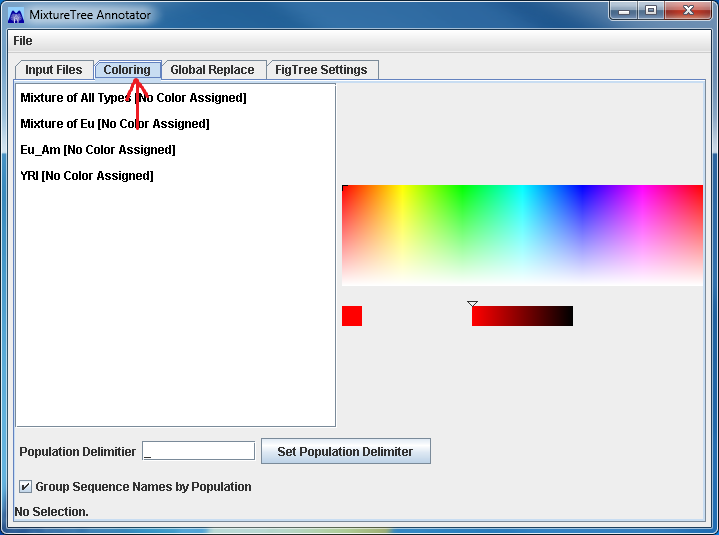


If the user clicks on the “Coloring” tab (shown by the red arrow), he or she will notice that there is “No Color Assigned” to any of the various categories. The other tabs are “Input Files”, “Global Replace”, and “FigTree Settings”. The “Input Files” tab is described on the preceding page. Information about “Global Replace” and “FigTree Settings” can be found in Appendix D – Advanced Panels. An understanding of how these two panels work is not required in order to use this program.

Step 2b: Loading Parameters [Optional]

This program supports the ability to save previous settings for later executions. A sample parameter file is provided for the example dataset. In order to open it, select **File -> Open Parameter File**.


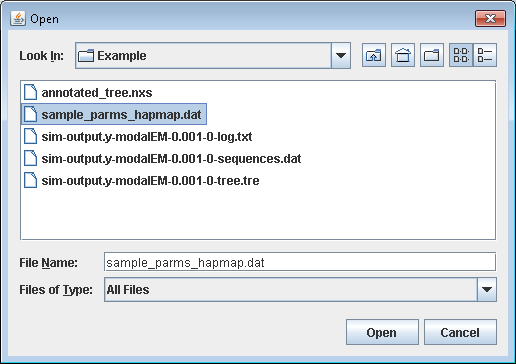


**Select the file “sample_parms_hapmap.dat” located in the “Example” folder, and click “Open”.** The coloring tab will immediately set the groups “Eu_Am” and “YRI” to blue and red respectively.

Step 3: Tree Coloring


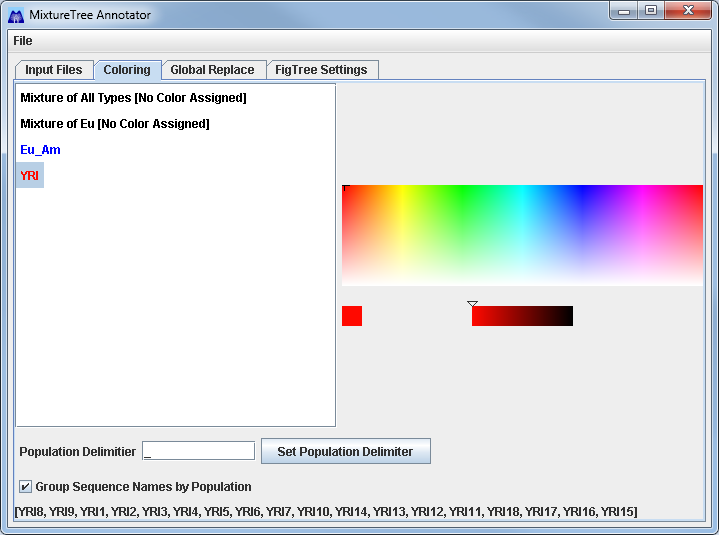


By going to the Coloring tab, the color that each label group will receive can be set. The final tree label(s) that belong to each group are listed in the status bar at the bottom as illustrated below.


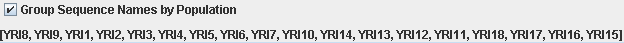


The above figure shows that labels that the color assigned to YRI will be applied to.

The user can select different colors by first selecting the label and then clicking on the color in the color picker on the right. The brightness of the color can be modified by choosing the appropriate brightness in the slider below. The color that will be applied is shown in a square box on the bottom left.


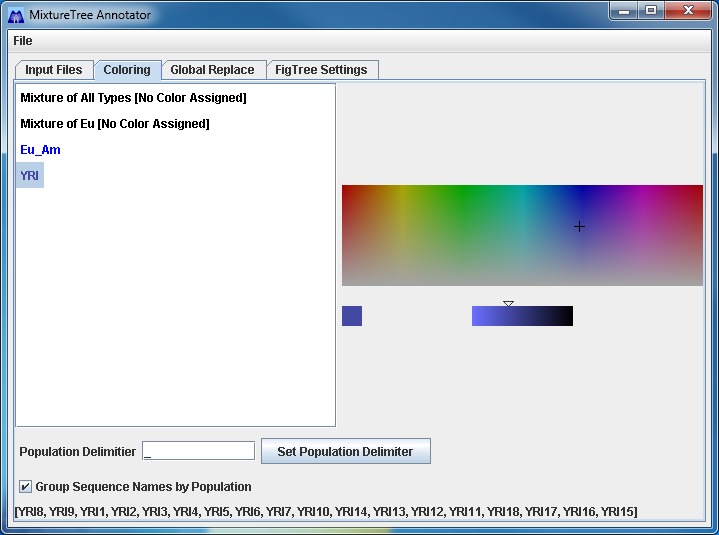


As an example, we have changed the color for the YRI group to a dark purple (formerly red). The user can also save the new parameters to a new file by going to **File->Save Parameter File** and selecting the new file to save.


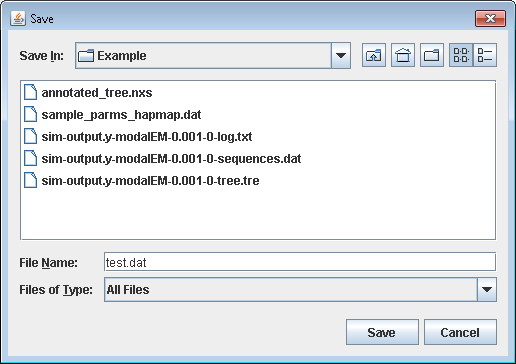


Step 4: Program Execution


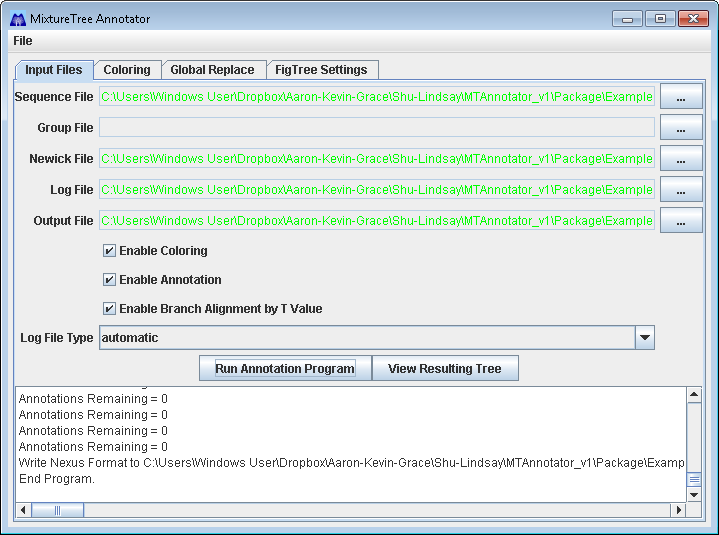


**Enable Coloring** – Using data provided in the “Coloring” tab each node will be properly colorized.

**Enable Annotation** – Using data provided from the “Log File”, each node will be properly annotated with the following information:

- Merge Time (t = *)
- Estimated Sequence (TCTCAT…)

**Enable Branch Alignment by T-Value** – This option requires that “Enable Annotation” was checked. Using the “Merge Time” data calculated, it will properly align each “t =” in the generated tree. See Appendix A for details.

In order to actually generate the final tree, ensure that the appropriate “Enable” checkboxes are enabled. **Then click “Run Annotation Program” and the program will generate the tree under the given output file. The program has completed when “End Program.” is displayed in the message window, and the text stops scrolling. In order to view the resulting tree, click on “View Resulting Tree,” and the annotation program will automatically open FigTree, as well as the generated tree.** If the user does not wish to use this functionality, the file will typically be found under the name “annotated_tree.nxs” in the path given by the “Output File” field. The customized version of FigTree can be opened by executing the file “figtree.jar” in a similar manner to executing the annotation program.

Step 5: Visualizing the resulting tree using FigTree


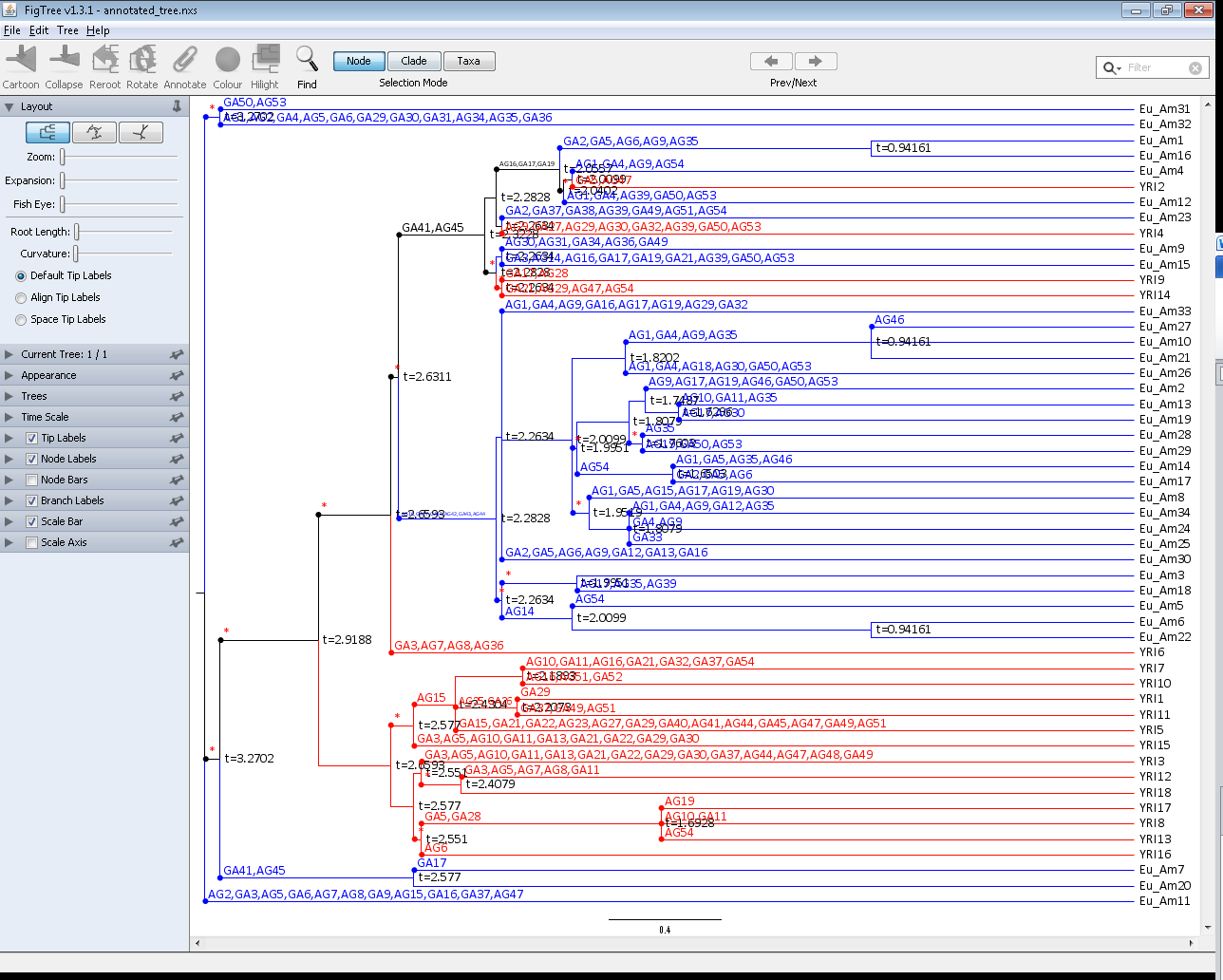


After opening the resulting output file with FigTree, the default screen looks similar to the above. The default screen may appear somewhat cluttered and difficult to read, especially if there are a large number of sequences. In order to remedy this, the user may use the “Zoom” and “Expansion” sliders located on the top-left. Note that the link to open FigTree by clicking the button “View Resulting Tree” in the current version of MixtureTree Annotator is only designed for Windows user. The Mac iOS or Linux users will have to open FigTree first, then the resulting trees can be viewed by FigTree.


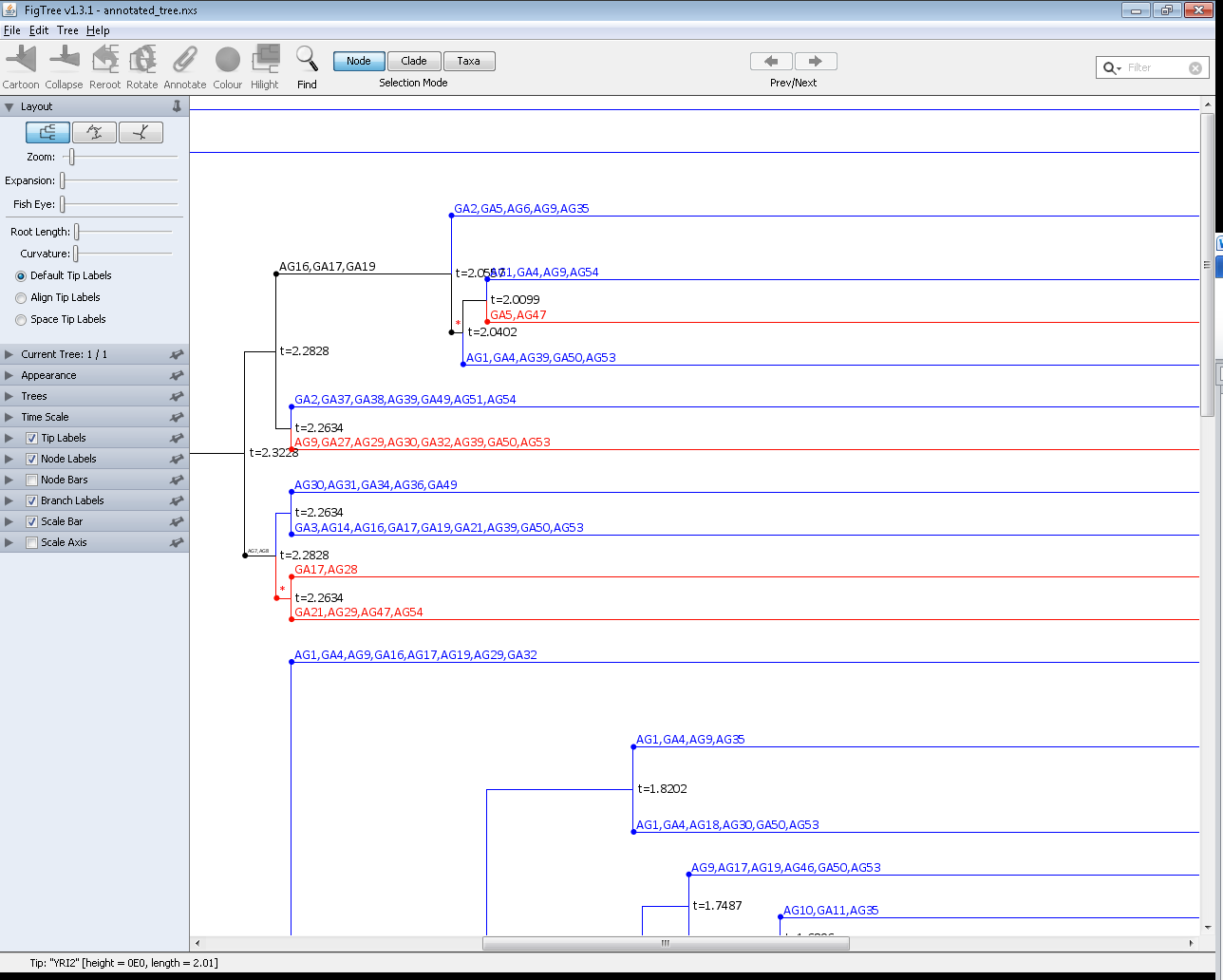


The figure above shows the formerly much cluttered top-left portion of the tree. By using the Zoom slider, the visibility of the tree has been greatly increased.

Step 6: FigTree Mutation Display


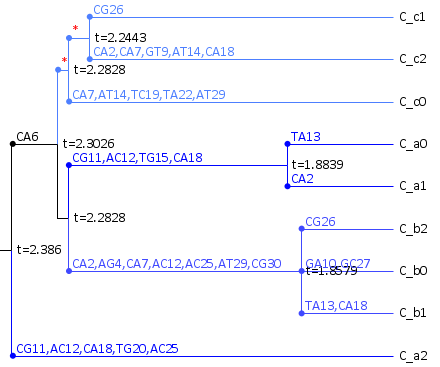


Figure 6-1: Basic Mutation Information.

The circle at the branch corner represents the point of mutation, and to the top right is written exactly what the mutation was. The mutation label follows the format given below. Each mutation is separated by a comma.

The mutation information follows the format xy### where x is the ancestral nucleotide at time t + ϵ, y is the mutated ancestor type at time t, and ### is the site at which the mutation occurred, with the first location starting at 1. ϵ must be positive. Note that the MixtureTree algorithm (Chen et al. 2011a) constructs the tree in reverse time. The current observed sequences is given by time = 0 and is located on the far right. The most recent common ancestor is located at the far left. At a given time point t, consider the following case.

| Sequence Location | 1 | 2 | 3 | 4 |
| --- | --- | --- | --- | --- |
| CAN at time t – 1 | A | C | T | G |
| CAN at time t | T | C | T | G |
| **Mutation Information** | AT1 | | | |

The mutation information for this case is AT1. That is at time t+ ϵ, where ϵ>0, the nucleotide of site 1 mutates from A to T. The merge time information is in a self-explanatory format. For example, according to Figure 6-1, C_c1 and C_c2 merged at approximately t=2.2443, using the time scale described in (Chen and Lindsay, 2006).

As a further example for clarification, CA6 in Figure 6-1 tells us that at time t=2.386, in site 6, the common ancestral sequence of two groups (C_c1, C_c2, C_c0, C_a0, C_a1, C_b2, C_b0, C_b1) and (C_a2) has nucleotide C, and the common ancestral nucleotide of (C_c1, C_c2, C_c0, C_a0, C_a1, C_b2, C_b0, C_b1) has nucleotide A in site 6. Similarly, we can compare this common ancestral nucleotide to C_a2 as performed on the following page.

| Sequence Location | 11 | 12 | 18 | 20 | 25 |
| --- | --- | --- | --- | --- | --- |
| Ancestral Nucleotide (C_c1, C_c2, C_c0, C_a0, C_a1, C_b2, C_b0, C_b1) and (C_a2) | C | A | C | T | A |
| C_a2 | G | C | A | G | C |
| **Mutation Information** | **CG11** | **AC12** | **CA18** | **TG20** | **AC25** |

In the above figure, there is a * instead of a mutation label. This means that the mutation label was too long to properly fit into the space provided. The mutation label can be found by clicking on the branch itself and viewing the status bar located in the bottom left.


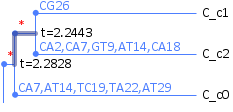


When the branch is properly clicked, it should appear highlighted as in the above figure.


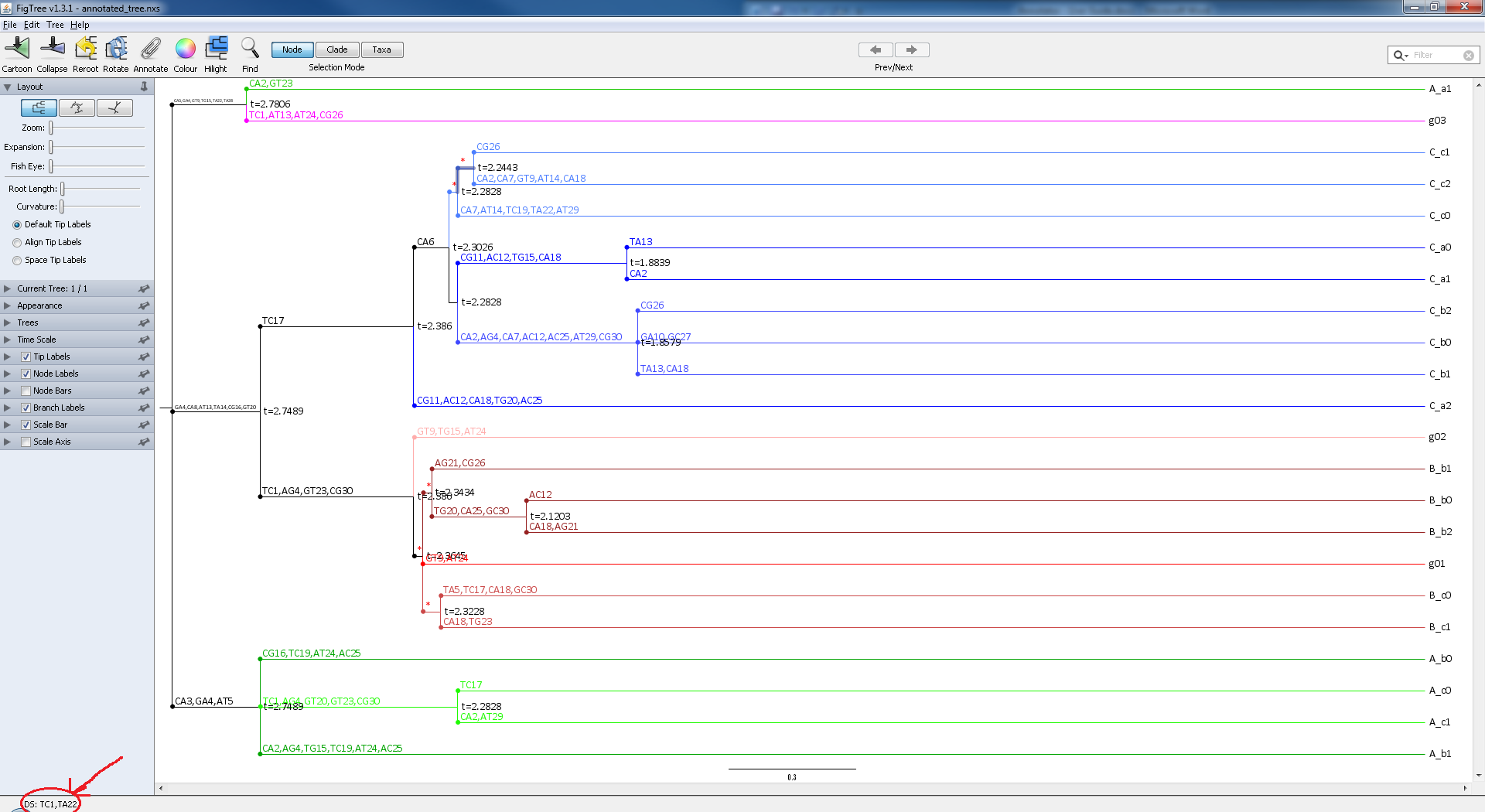


The status bar will appear similar to the above figure (circled in red).

**Appendix A – Advanced Annotation Features**

Option: Enable Branch Alignment by T-Value

| Option Disabled | **Option Enabled (Default)** |
| --- | --- |
| 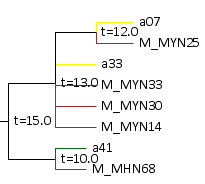 | 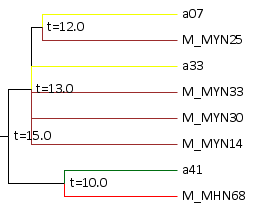 |

MixtureTree with Extended Models (Chen *et al.*, 2012) generates a tree in which the “t=” values are not aligned properly. Note how t = 13.0 and t = 10.0 are both aligned on the y-axis and t = 12.0 is to the right of t = 10.0. In the figure on the right side, note how with this option, all values are correctly aligned, and progress from a larger value of “t” on the left-hand side to a smaller value of “t” on the right-hand side.

**Appendix B – Advanced Coloring Features**

Colorization Priority

- If a group contains only M_*, the color assigned to that group will be the color for “Mixture of M” as given on the “Coloring” panel.
- If a group contains only Y_*, the color assigned to that group will be the color for “Mixture of Y” as given on the “Coloring” panel.
- If a group contains both M_* and Y_*, the color assigned to the group will be the color for “Mixture of All Types” as given on the “Coloring” panel.
- If a group contains only M_MHN, the color assigned is the color for M_MHN.

Population Delimiter (Colorization)


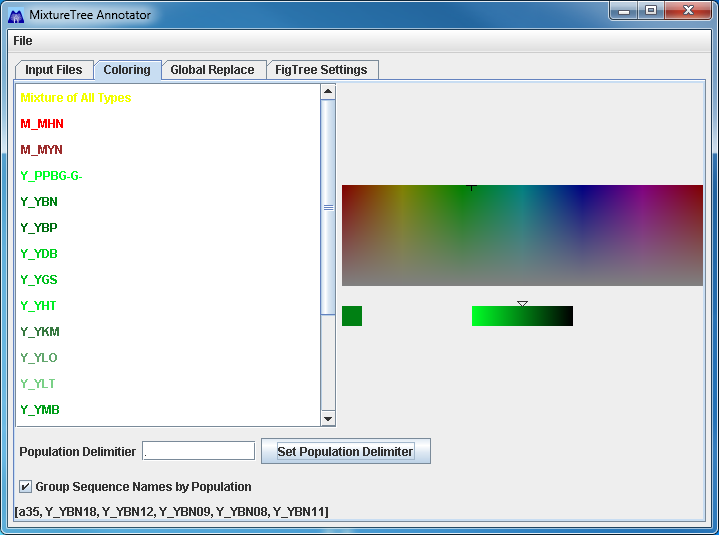


The population delimiter tells the program the appropriate symbol that differentiates between the ethnicity names and the population names. In the above example, the appropriate delimiter is an underscore character (_). By replacing the _ with a dot (.) and clicking on “Set Population Delimiter”, one notices that the “Mixture of M” and “Mixture of Y” disappears, as the program no longer recognizes these as being ethnicity names. In this case, “Mixture of M” and “Mixture of Y” represent the groups of sequences that consist of different populations in the same ethnicity which had the same genetic sequence.

Option: Group Sequence Names by Population (Colorization)


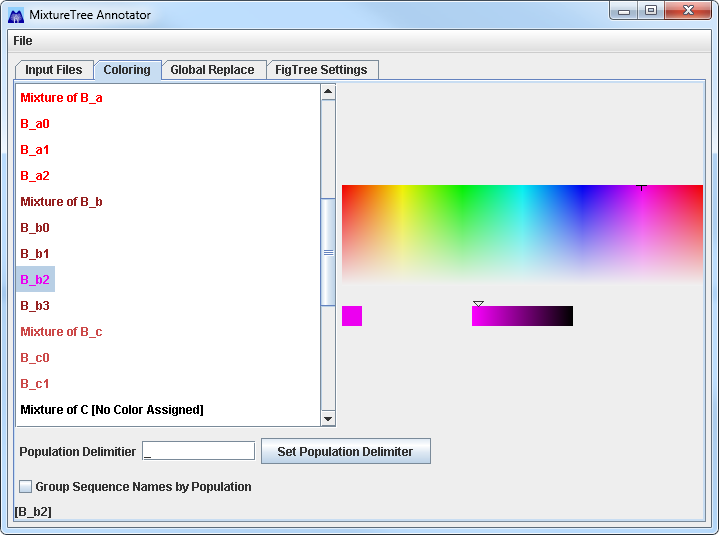


By disabling the option to Group Sequence Names by Population, the colorization program provides the opportunity to individually color certain labels. In the example below, B_b2 will be colored differently from the rest of the B_b nodes.

**
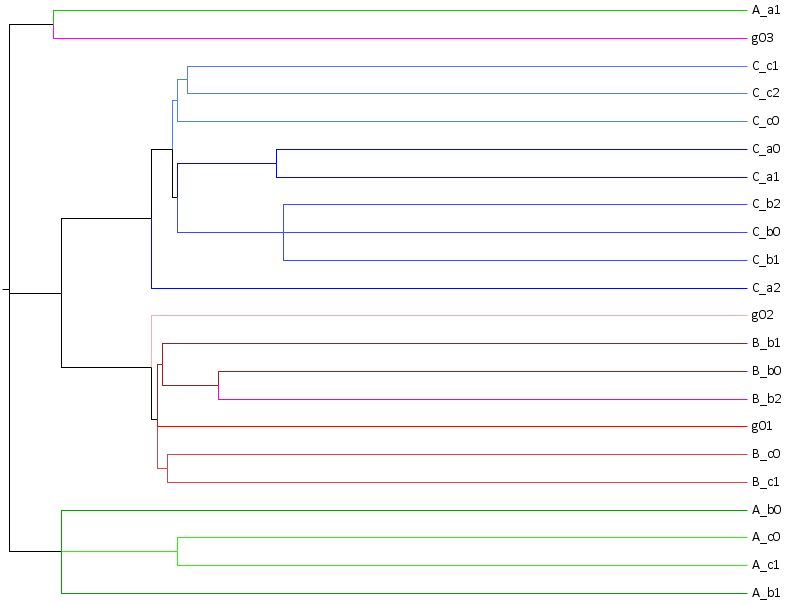
**

**Appendix C – File Format Descriptions**

**Sequence File** – This file contains the sequence data for this program. The sequence data should be in the following format:

<Sequence Name> <Sequence Data> <Frequency of Occurrence>

The sequence data must be in uppercase.

Example: Y_YYM28 TCTCATGAGCAG 2

**Group Membership File** – This file contains the group definitions. The data should be in the following format:

<group name>=<member>,<member>,…,

The group name can only contain the characters A to Z, a to z, underscore (_), and minus (-).

Example: a03=M_MHN04,M_MHN17,M_MHN82,M_MYN36,Y_YHT06,Y_YHT08,Y_YKM11,Y_YKM28,Y_YLO31,

**Newick File** – This file contains the generated Newick Tree. The data should be in standard Newick format.

<http://en.wikipedia.org/wiki/Newick_format>

Example: (A:0.1,B:0.2,(C:0.3,D:0.4):0.5);

**Log File** – This file contains the generated EM algorithm output file. There are two supported types of log files which are detailed below in Log File Type.

No Example Provided.

**Log File Type** – (automatic, mixturetree, extendedmodel, mif)

Automatic – Try to automatically determine what type the given log file is. If a type cannot be determined, “extendedmodel” is chosen.

MixtureTree – Log file generated by Bernoulli Mixture Models (MixtureTree v3.0)

ExtendedModel – Log file generated by Extended Models (MixtureTree v3.0)

mif – Log File generated by both models in MixtureTree v3.0b

Mixture Tree – The following lines are used to calculate the required data:

- When p equals 0.06, T = 0.12783, mutated at loci 18 in cluster 86
- 1 111010101010010100 0.008928571429 1 Seq-1-0

Extended Model – The following lines are used to calculate the required data:

- 2012-03-02 07:04:56 Y_YYM28,TCTCATGAGCAGATGTACACAGTATGACTC…(truncated)…,2
- 2012-03-02 07:04:56 1 TCTCATGAGCAGATG(…truncated…) 0.003724394786 2
- 2012-03-02 09:12:52 t=2 after EM steps
- 2012-03-03 12:45:26 Merge cluster 94(Y_YMI19) and 266(Y_MHN103) TCTCATGAGCA…(trunc)

**Output File** – This file will be written using the Nexus Format, with a few extensions that are specific to FigTree.

<http://en.wikipedia.org/wiki/Nexus_file>

<http://code.google.com/p/beast-mcmc/wiki/NexusMetacommentFormat>

No Example Provided.

**Appendix D – Advanced Panels**


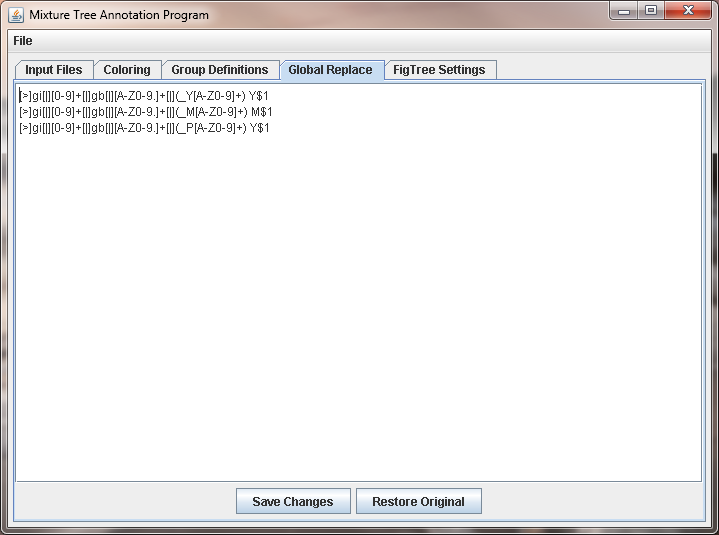


The “Global Replace” panel is intended for batch renaming of different node labels. Each line is divided by a space, and follows the format for a regular expression. The regular expression is followed the rule of pattern matching developed by Java^TM^. The left side is the pattern to match, and the right side is the pattern to replace it with. There can only be one space per line, and the space must divide the left and right side.

From the expressions in the above figure, the following will be replaced.

| **Old Sequence Name** | **New Sequence Name** |
| --- | --- |
| >gi\|47079470\|gb\|AY546762.1\|_MHN35 | M_MHN35 |
| >gi\|47079575\|gb\|AY546867.1\|_PPBG-G-21 | Y_PPBG-G-21 |
| >gi\|47079700\|gb\|AY546992.1\|_YKM26 | Y_YKM26 |

The tutorial for Java regular expression can be found in the link below:

<http://docs.oracle.com/javase/tutorial/essential/regex/>


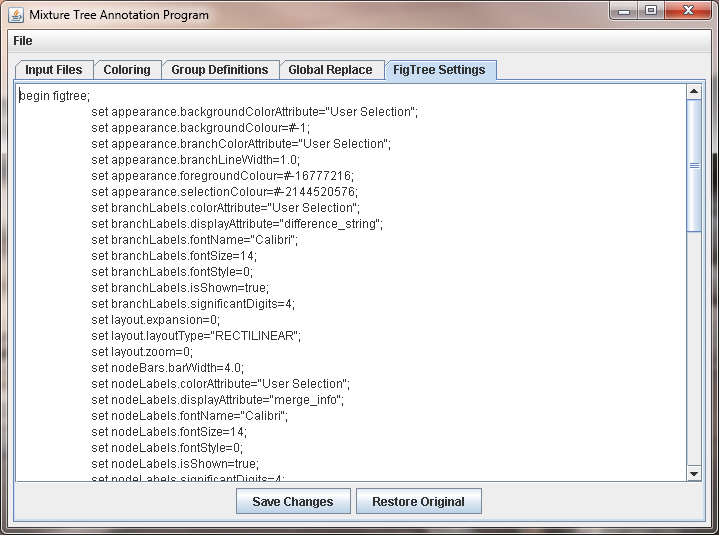


The “FigTree Settings” panel is intended to set the default view in FigTree. User should never have to modify these settings but are provided for convenience.

**Appendix E – FigTree Customizations**

FigTree Website: <http://tree.bio.ed.ac.uk/software/figtree/>

The patch figtree_custom_v4_to_svn_150.patch is intended to patch SVN REVISION 150 only. Instructions to obtain this version of the source code may be found here: <http://code.google.com/p/figtree/source/checkout>.

Change List

- Space Tip Labels was added so that labels do not line up when the option is checked. Instead, each label is given a rotating offset so that the text of one label does not run into other labels when the font size is too large. This is intended to make it possible for larger graphs to fit onto a standard sheet of paper while keeping the node labels visible.
- When a branch label is too long, the window dimensions are no longer re-sized to accommodate for this. This is intended to prevent the graph from moving when Branch Labels becomes checked/unchecked. This change is applicable to all choices of “Display” for Branch Labels.
- With the above point, as a branch label that is too long will no longer be able to be read, all branch labels that do not fit on the line given will be replaced with a *. This is applicable only to when the Branch Labels is set to display “difference_string”. In order to read the contents of the branch label, one has to click on the branch with the label one wishes to read, and view the status bar. It will be the case that “difference_string” will show in the status bar regardless of if the user has “difference_string” selected or not.
- A dot is displayed on the branch where a mutation occurred to provide better ability to visualize the tree.
- Fixed bug where “Line Width” spinner would not be initially set to the correct value and where it would ignore the saved “Line Width” value.
- Printing feature was disabled in FigTree code as it does not work with the “Branch Labels” change. In order to work around this, use Export to PDF … and then print the resulting PDF with a PDF Viewer.

Known Bugs

- Do not switch the display mode from Rectilinear or the program will crash.

**References**

Chen, S. C., Li, M., Rosenberg, M., and Lindsay, B (2011) ”Mixture tree construction and its applications”. *Handbook of Computational Statistics: Statistical Bioinformatics*. Eds. Lu Henry, Bernhard Schölkopf, and Hongyu Zhao. Springer-Verlag, **147-135**.

Chen,S.C. and Lindsay,B. (2006) Building mixture trees from binary sequence data. *Biometrika*, **93(4),** 843-860.

Chen S.C. and Lindsay B. (2012) Improving mixture tree construction using better EM algorithms. *Comput. Stat. Data Analysis*. **In Press**.

Chen S.C., Rosenberg M., Lindsay B. (2011) MixtureTree: a program for constructing phylogeny. *BMC Bioinformatics*, **12**, 111+.

Chung Y, Lindsay B, Chen SC. (2012) Creating Likelihood-enhanced Density Estimators with the EM Algorithm. **Manuscript**.

Rambaut, A (2007) FigTree [http://tree.bio.ed.ac.uk/software/figtree/].
